# Mental health status of nurses working in private and public sectors

**DOI:** 10.1192/j.eurpsy.2025.1589

**Published:** 2025-08-26

**Authors:** N. Rmadi, A. Hrairi, M. Hajji, F. Dhouib, F. Ben Atia, I. Ben Hnia, M. Hajjaji, K. Jmal Hammami

**Affiliations:** 1 Occupational medicine department, Hedi chaker university hospital, University of Sfax; 2 Family medicine department; 3 Higher Institute of Nursing Sciences of Sfax, university of Sfax, Sfax, Tunisia

## Abstract

**Introduction:**

Nurses are at the forefront of patient care and are often exposed to various stressors that can impact their mental health. The mental health of nurses working in both the private and public sectors is a significant issue that requires attention.

**Objectives:**

In this study, we propose to compare psychological distress of nurses working in private and public sectors.

**Methods:**

It was a cross sectional study conducted among 200 nurses (100 from each of the private and public sector). We used the 6-item Kessler scale (K6) to measure symptoms of psychological distress.

**Results:**

The average age of nurses working in the public sector was 33.24 ± 9.34 years versus 31.93 ± 7.68 years in the private sector. Most staff (33.5%), whether in the public or private sector, had professional seniority of between 6 and 10 years. The average K6 score was significantly higher in the public sector (9.01) compared to the private sector (7.35) (p-value = 0.01). The likelihood of experiencing psychological distress was lower in the private sector compared to the public sector (p= 0.034; OR= 0.37; 95% CI [0.14-0.95]).

**Conclusions:**

These findings highlighted potential implications for the mental health of nurses based on their employment sector, showing that prolonged daily stress could have a negative impact on their psychological well-being.

**Disclosure of Interest:**

None Declared

